# Modelling acute leukemias in mice: clonal evolution and the emergence of leukemic stem cells

**DOI:** 10.1186/1753-6561-7-S2-K1

**Published:** 2013-04-04

**Authors:** Bastien Gerby, Trang Hoang

**Affiliations:** 1 Institute of Research in Immunology and Cancer, University of Montréal, Montréal, Québec, Canada H3C 3J7; 2 Molecular Biology Program, University of Montréal, Montréal, Québec, Canada H3C 3J7; 3 Departments of Medicine, University of Montréal, Montréal, Québec, Canada H3C 3J7; 4 Department of Pharmacology. University of Montréal, Montréal, Québec, Canada H3C 3J7; 5 Department of Biochemistry, University of Montréal, Montréal, Québec, Canada H3C 3J7

## 

The concept of cancer stem cells (CSCs) is based on a hierarchic model of cancer whereby cells within a tumour exhibit distinct biological characteristics and only CSCs are able to grow indefinitely and to maintain the neoplastic process. The molecular and cellular characteristics of CSCs are thought to be due to genetic and epigenetic states reminiscent of those normal stem cells. Precisely, cancer arise from the neoplastic transformation of stem cells or committed-progenitor cells [1] through two types of events. First, normal stem cells can acquire genetic alterations that alter its growth control, increase its resistance to apoptosis and interfere with cell differentiation. Second, non-stem cells can be altered by the oncogenic process to reacquire the self-renewal properties of normal stem cells. In both cases, these characteristics confer a particular resistance to drugs, implying that CSCs are involved in the persistence of tumour cells during treatment and consequently, are responsible of relapses.

The concept of CSCs has come from pioneering studies on acute myeloid leukemia (AML) which have defined a distinct subpopulation of tumour cells, the leukemia initiating cells (LICs), characterized by their capacity to initiate the disease when transplanted into imuno-deficient mice [2,3]. A confounding issue in the field has been the equation of CSCs with the cell of origin of acute leukemias. Indeed, these original studies and subsequent work suggested that AML derived from the malignant transformation of hematopoietic stem cells (HSCs) [4,5]. However, growing evidence based on cellular and molecular studies led to the recognition that the cell of origin of AML is a committed progenitor that normally lack any potential for self-renewal [6,7]. This controversy may be reconciled by assuming that AMLs may represent in some cases a stem cell disorder, while in other cases, the reacquisition of stem cell characteristics by a committed progenitor [8].

The situation is different in paediatric acute lymphoblastic leukemia (ALL), where there is evidence that leukemia initiating activity is observed not only in the immature cell population but also in populations corresponding to a range of normal precursor cells [9,10]. Precisely, the analysis of leukemic and pre-leukemic stem cell populations in a pair of identical twins indicate that the putative stem cell responsible for initiating and maintaining B-ALL are not a fixed cell identity but evolve both in genotype and phenotype [11]. Comforting this observation, a process of clonal evolution at the level of LIC populations was provided both in B-ALL and T-ALL. Indeed, the molecular investigation of individual LIC helped to establish a complex clonal architecture of individual leukemia, showing that LIC are genetically heterogeneous due to the process of clonal evolution [12-14].

T-acute lymphoblastic leukemia (T-ALL) represents about 15% of paediatric leukemias. Several studies in these last years have, in part, elucidated the molecular mechanism of T-ALL transformation. Indeed, T-leukemogenesis is a multi-step process characterized by the acquisition of several oncogenic events. Especially, genes encoding the SCL transcription factor and its nuclear partners LMO1 and LMO2 are frequently deregulated in T-ALL. Furthermore, activating mutations of *NOTCH1* are found in more than 50% of T-ALL cases, and are frequently associated with chromosomal abnormalities in the *SCL* and/or *LMO1/2* locus [15,16], implying that these mutational events frequently collaborate during neoplastic transformation of thymocytes. It was originally proposed that the phenotype of the tumor reflects the cell of origin of T-ALL [17]. However, recent studies indicate that fully transformed T-leukemic cells are functionally heterogeneous and may originate from the leukemic transformation of an early T-cell progenitor [18,19]. Furthermore, it has been recently shown that the overexpression of the *LMO2* oncogene in the thymus induce the emergence of a pre-leukemic stem cell (pre-LSC) population [20] but the identification of the cell of origin of T-ALL and the mechanisms by which these oncogenes reprogram normal thymocytes to become T-LIC remain unclear. We took advantage of a transgenic mouse model that closely reproduces paediatric T-ALL to define oncogenic events during the pre-leukemic phase.

We show that SCL-LMO1 inhibit thymocyte differentiation at the double negative to double positive transition, via inhibition of two transcription factors that are essential in the thymus, HEB and E2A [21,22]. Moreover, SCL-LMO1 reprograms thymocyte progenitors to confer abnormal self-renewal capacity. The acquisition of stem cell-like properties establishes a pre-leukemic state in thymocytes by causing an expansion of the CD4^-^CD8^-^ double negative (DN) population of progenitors that actively proliferate under the influence of the pre-TCR and are therefore at risk of acquiring mutations. Strikingly, our data indicate that the pre-TCR favors the acquisition of *Notch1* mutations in *SCL-LMO1* pre-leukemic stem cells [23]. Finally, *SCL*, *LMO1* and *NOTCH1* together induce a polyclonal disease in transgenic mice, which is comparable to that induced by transplantation of a single leukemic stem cell. We therefore conclude that these three oncogenes are sufficient to transform DN thymocytes.

In summary, we show that in T-ALL, the target cell of transformation by the SCL-LMO1 oncogenes are double negative thymocytes that acquire aberrant self-renewal activities but remain non-leukemogenic. Acquisition of activating *Notch1* mutations then transforms these thymocyte progenitors into leukemic stem cells [23].

## Competing interests

There are no competing interests in this presentation.
